# Supplementary material for: Downscaling Industrial-Scale Syngas Fermentation to Simulate Frequent and Irregular Dissolved Gas Concentration Shocks
Source: Bioengineering (Basel). 2023 Apr 25;10(5):518. doi: 10.3390/bioengineering10050518 (PMC10215885; doi:10.3390/bioengineering10050518)
Supplement: Supplementary file 1 [file bioengineering-10-00518-s001.zip › Supplementary information.pdf]

Supplementary information for:

Downscaling industrial-scale syngas fermentation to simulate frequent and irregular dissolved gas concentration shocks

Lars Puiman[1], Eduardo Almeida Benalcázar[1], Cristian Picioreanu[2], Henk J. Noorman[1,3], Cees Haringa[1]

1: Department of Biotechnology, Delft University of Technology, Delft, The Netherlands

2: Biological and Environmental Sciences and Engineering Division, King Abdullah University of Science and Technology, Thuwal, Saudi Arabia

3: Royal DSM, Delft, The Netherlands

Corresponding author: Cees Haringa

Address: Delft University of Technology, Faculty of Applied Sciences, Department of Biotechnology, Van der Maasweg 9, 2629 HZ, Delft, The Netherlands

Email: C.Haringa@tudelft.nl

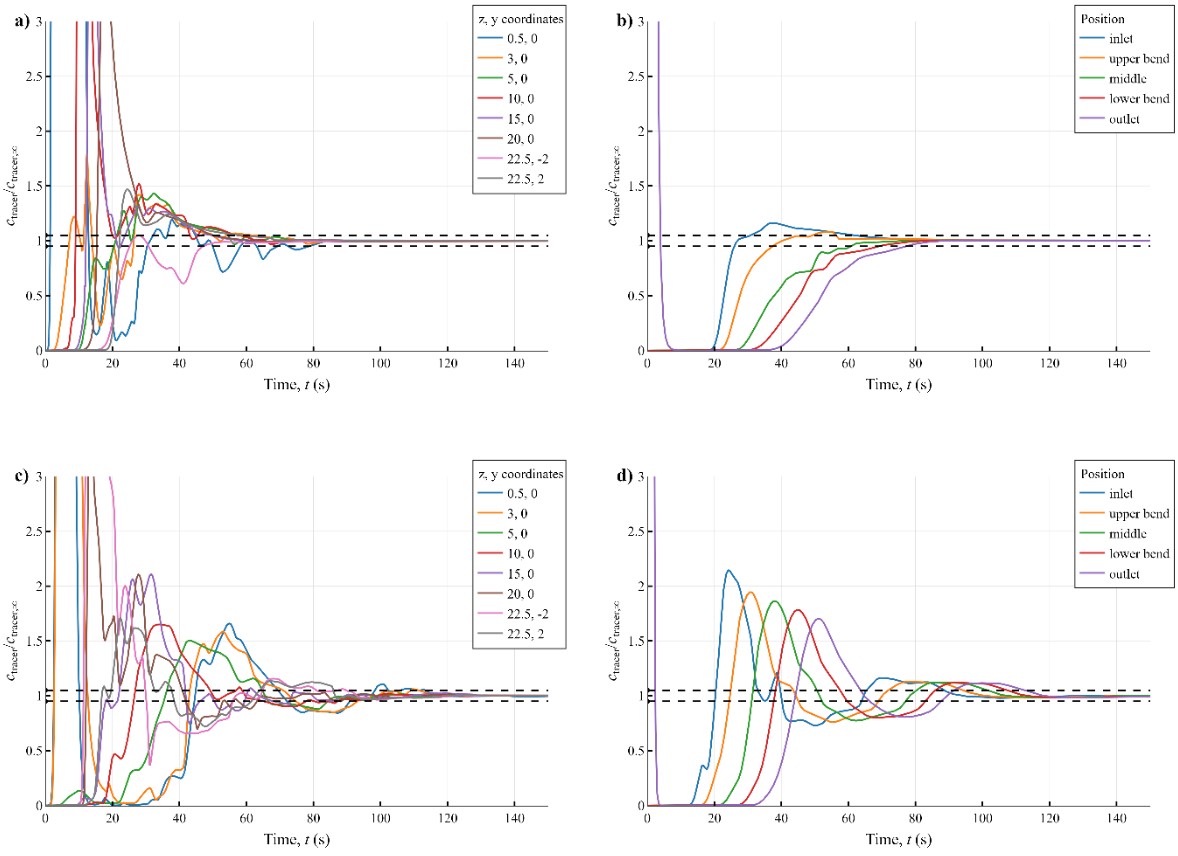

Figure S1. Mixing study in the EL-GLR. A tracer with the same properties as the fluid phase was injected at the outlet of the downcomer and its normalized concentration recorded at several positions in the riser (a, c – coordinates in m) and downcomer (b, d). The 95% mixing time t_m is obtained when all curves are within the dashed lines representing a 5% deviation around the final tracer concentration. t_m was recorded for a case without reaction (only the hydrodynamic model was enabled) (a, b) and a case including mass transfer and reaction at 25 g L^{-1} biomass without CO_2 production (c, d). See (Puiman et al., 2022) for more details about the hydrodynamic model and the reactor geometry.

t_m without reaction is around 80 s, while with reaction it is around 115 s. This difference is explained by the lower gas hold-up in the case with reaction since the syngas is consumed. The circulation time t_c is estimated from the mixing time as $t_m \sim$ 3-5 t_c (van't Riet and van der Lans, 2011).

Table S1. Characteristics of the scale-down simulator (Garcia-Ochoa and Gomez, 2009; Zanghi et al., 2017).

**Reactor type and dimensions**	Applikon 3L Glass bioreactor
Volume reactor (L)	3
Height (m)	0.234
Diameter (m)	0.13
Impeller diameter, d_i (m)	0.06
Power number, N_{Po}	1.5
Liquid volume, V_L (L)	2
**Mass transfer parameters**	
α	0.783
β	0.459
**Fixed operating conditions**	
Temperature (°C)	37
Pressure (kPa)	101
pH	5 (for solventogenesis)
Ethanol concentration (g L^{-1})	50 (if possible because of inhibition)
Acetate concentration (g L^{-1})	12
Dilution rate (h^{-1})	0.021
Gas flow rate (vvm)	0.05

Statistical analysis of lifelines

It was determined how many lifelines were required to obtain statistically independent probability density distributions of the residence time in peaks and valleys. This was done by calculating the Kullback-Leibler divergence D_{KL} (Equation S1) or the relative entropy for the variables of interest (e.g. the residence time distribution in peaks and valleys for CO and H_2). D_{KL} is a common tool in data sciences to measure dissimilarity between two probability distributions (Bishop, 2006). By minimizing D_{KL} of a distribution with respect to a given ("true") distribution, it can be determined how close a distribution is to the true distribution (Akaike, 1998; Bishop, 2006). As a "true" distribution (with $N_p, N_{t_c} \to \infty$) was not available, it was assumed that the distribution obtained with all peaks and valleys, $P\left(N_p N_{t_c}\right)_\infty$, is sufficiently representative of the "true" distribution.

$$D_{KL}\left(P\left(N_p N_{t_c}\right)_\infty \parallel P\left(N_p N_{t_c}\right)\right) = \sum_{i=1}^{k} P_i\left(N_p N_{t_c}\right)_\infty \ln\left[\frac{P_i\left(N_p N_{t_c}\right)_\infty}{P_i\left(N_p N_{t_c}\right)}\right] \tag{S1}$$

From the analysis became clear that with $N_p N_{t_c} \sim O(10^5)$ the D_{KL} approaches zero (Figure S2) for the probability distributions describing the duration of a peak in CO concentration. One could argue that even around $N_p N_{t_c} \sim O(10^4)$ the information gain by extending the set of lifelines is small. These results give an indication of the required number of particles and runtime for lifeline analysis of environmental fluctuations, which is considerably lower than the numbers typically applied for such analysis (e.g. $N_p N_{t_c} > O(10^6)$ (Haringa et al., 2016; Kuschel and Takors, 2020; Siebler et al., 2019)). As an exception, McClure et al. (2016) only used $N_p N_{t_c} \sim O(10^4)$ while obtaining statistically satisfactory results. It must be noted that our analysis is only applicable when particles act as passive tracers. When the simulated biomass particles affect the Eulerian flow or concentration field (e.g. two-way coupling with metabolic models) (Haringa et al., 2018; Lapin et al., 2004), a higher N_p is required to achieve a sufficiently homogeneous spatial biomass distribution (Haringa et al., 2017). In syngas fermentation modelling, this could be applicable if one wants to study the effect of local microbial CO_2 production or consumption, using a detailed kinetic metabolic model.

By calculating D_{KL}, we saved a significant amount of simulation and data processing time: the analyses with 10 and 25 g L^{-1} (650 s and 40,000 particles) only required 20 GB of data instead of 200 GB with the 5 g L^{-1} case (1000 s and 160,000 particles). Next to checking whether the Lagrangian averages matches the Eulerian average (Siebler et al., 2019), which is – in case of sufficient mixing – usually after 3 mixing times per particle, we suggest calculating D_{KL} as well to check variability of the results.

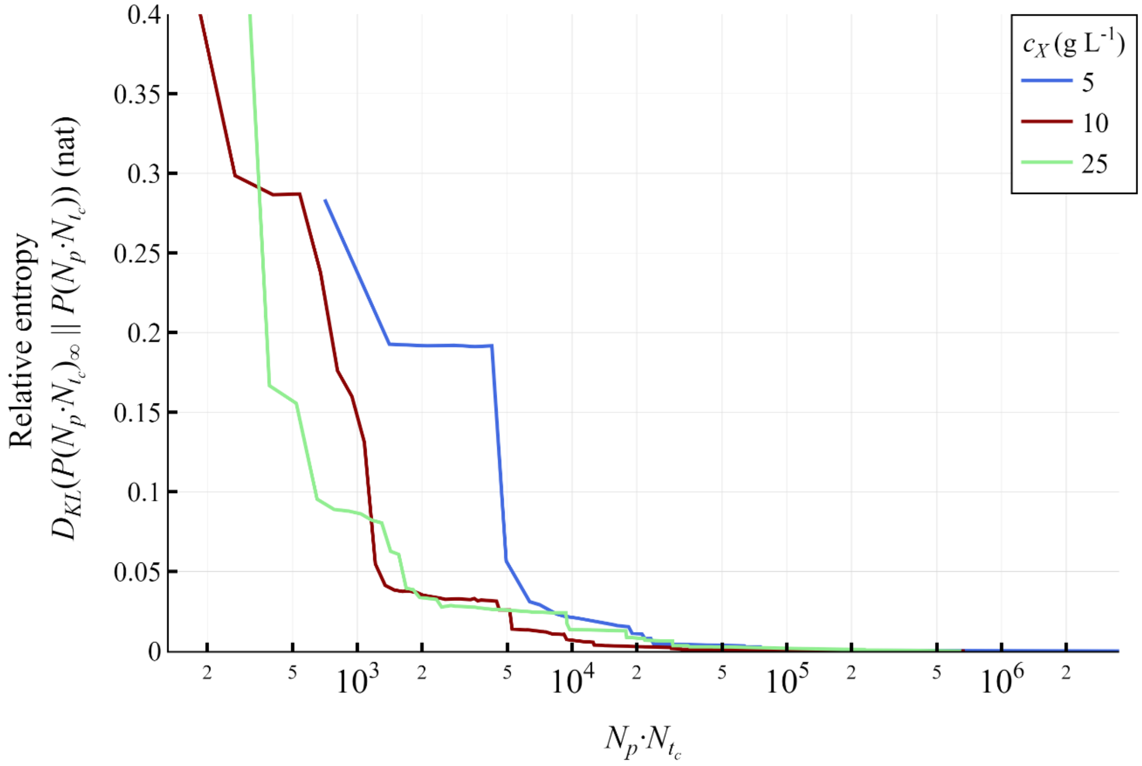

Figure S2. Kullback-Leibler divergence D_{KL} determined using an increasing $N_p N_{t_c}$ of the residence time distribution in a CO peak. Blue: D_{KL} obtained from distributions derived from lifelines with 5 g L^{-1} of biomass with $\left(N_p N_{t_c}\right)_\infty \sim 3 \cdot 10^6$. Green: 10 g L^{-1} and $\left(N_p N_{t_c}\right)_\infty \sim 6 \cdot 10^5$, Red: 25 g L^{-1} and $\left(N_p N_{t_c}\right)_\infty \sim 6 \cdot 10^5$.

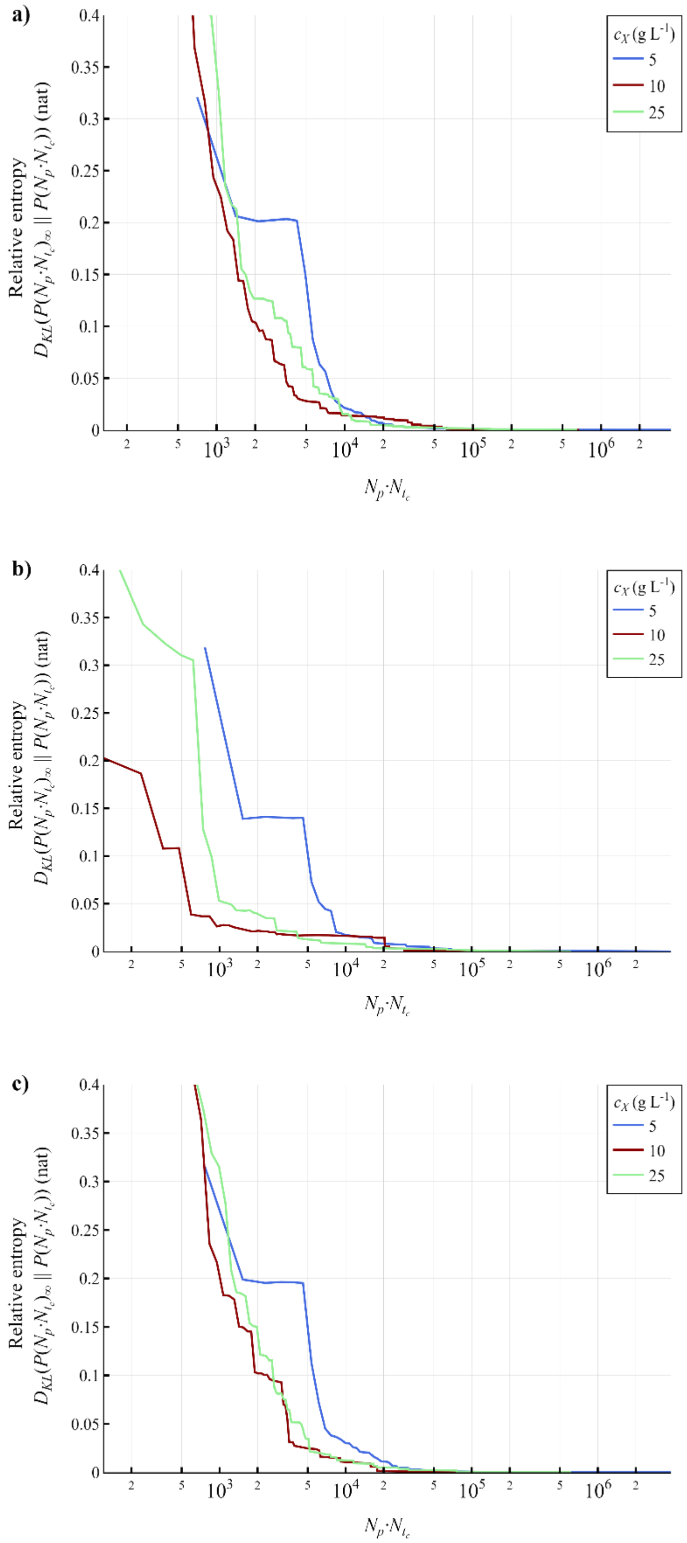

Figure S3. Kullback-Leibler divergence D_{KL} determined using an increasing $N_p N_{t_c}$ of the residence time distribution a) in a CO valley, b) H_2 peak, and c) H_2 valley. Blue: D_{KL} obtained from distributions derived from lifelines with 5 g L^{-1} of biomass with $\left(N_p N_{t_c}\right)_\infty \sim 3 \cdot 10^6$. Green: 10 g L^{-1} and $\left(N_p N_{t_c}\right)_\infty \sim 6 \cdot 10^5$, Red: 25 g L^{-1} and $\left(N_p N_{t_c}\right)_\infty \sim 6 \cdot 10^5$.

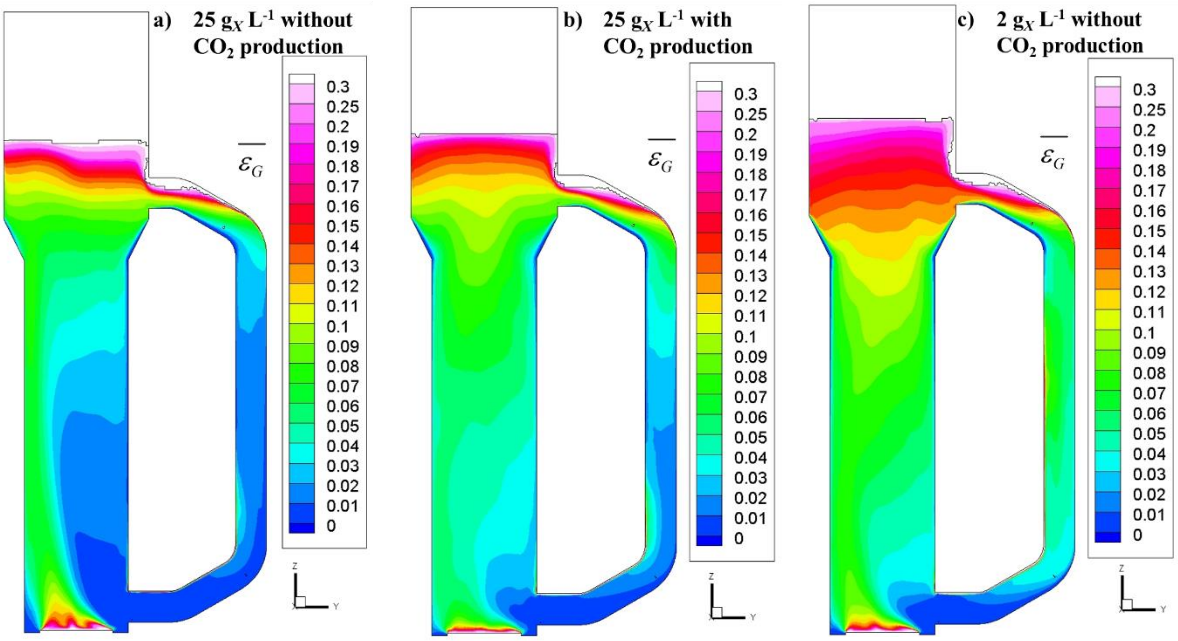

Figure S4. Surface plots of the 200 s time-averaged gas hold-up in the zy-plane (x = 0) of the EL-GLR, obtained a) with 25 g L^{-1} biomass without including CO_2 production, b) 25 g L^{-1} biomass while including CO_2 production and c) 2 g L^{-1} biomass without CO_2 production.

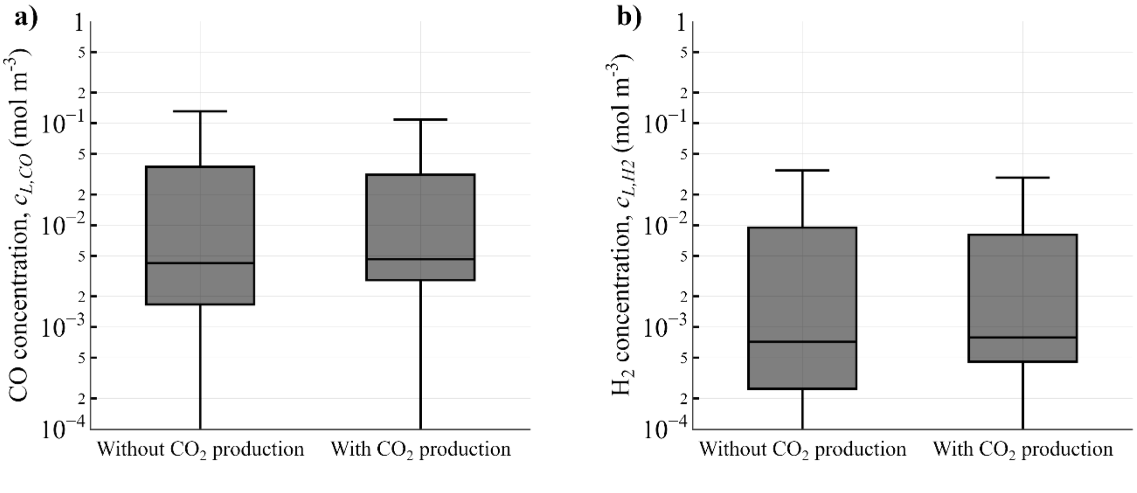

Figure S5. Variation of a) dissolved CO and b) dissolved H_2 concentrations within the EL-GLR reactor volume in the cases without and with CO_2 production (with 25 g L^{-1} biomass). In the boxplot each quartile represents 25% of the dispersion volume with a specific concentration.

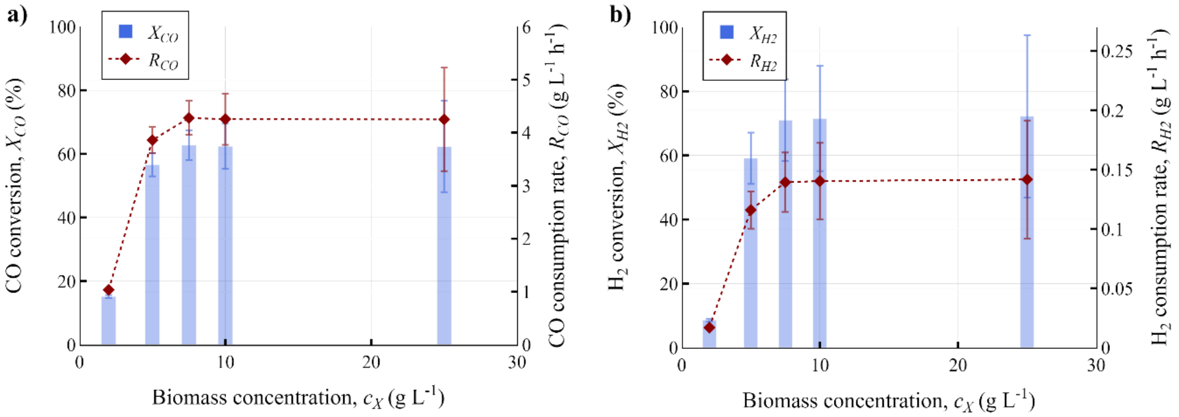

Figure S6. For the varying biomass concentrations the Eulerian results for a) CO and b) H_2 conversion (blue bars) and consumption rates (red markers) are given.

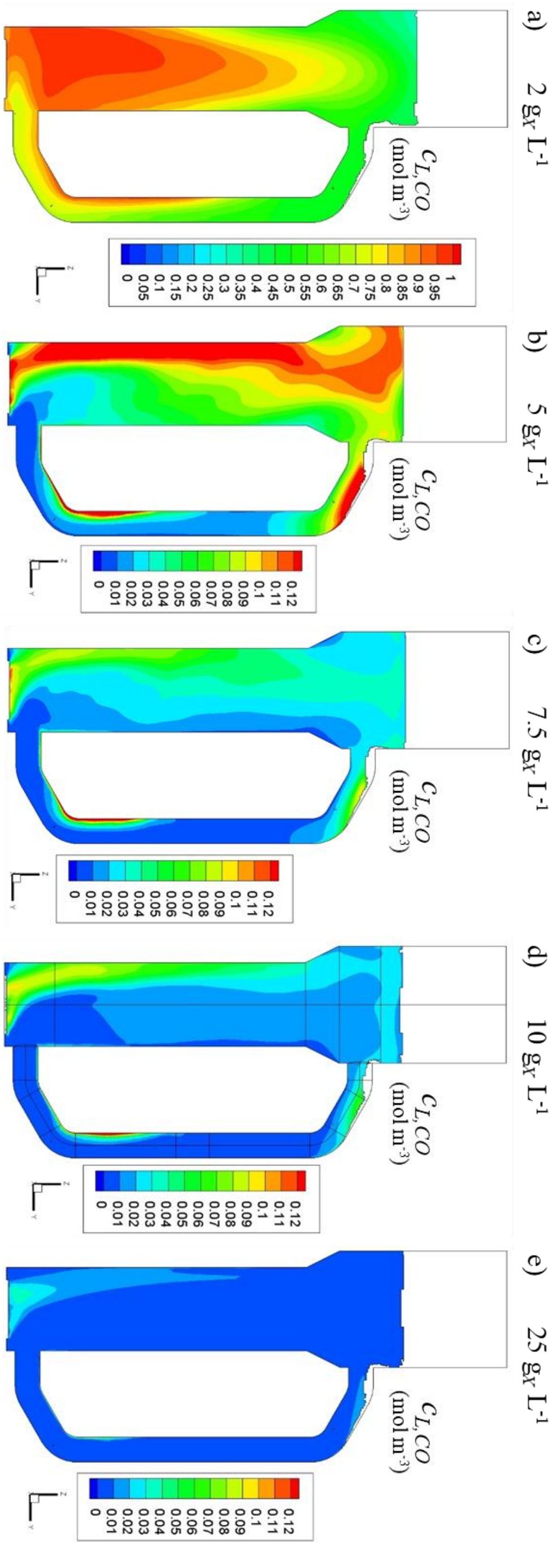

Figure S7. Surface plots of the dissolved CO concentration in the zy-plane (x = 0) of the EL-GLR, for several concentrations of biomass (2, 5, 7.5, 10, 25 g L^{-1}) (a-e).

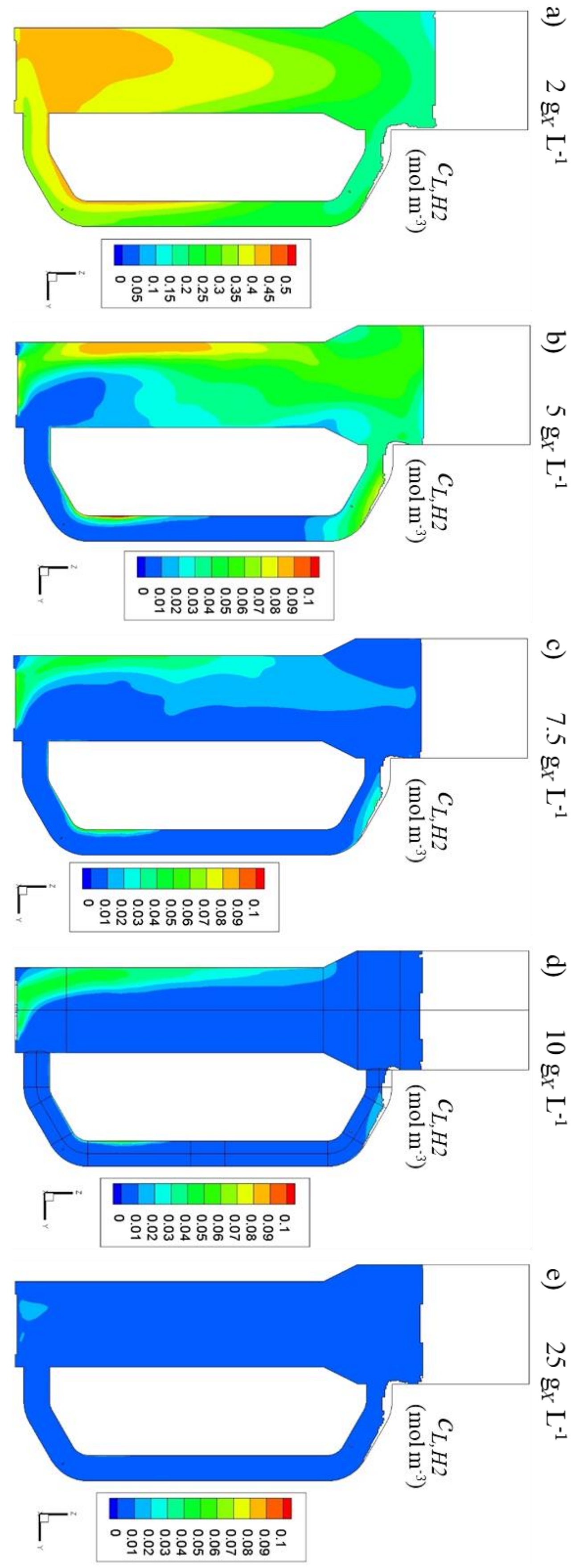

Figure S8. Surface plots of the dissolved H_2 concentration in the zy-plane (*x* =0) of the EL-GLR, for several concentrations of biomass (2, 5, 7.5, 10, 25 g L^{-1}) (a-e).

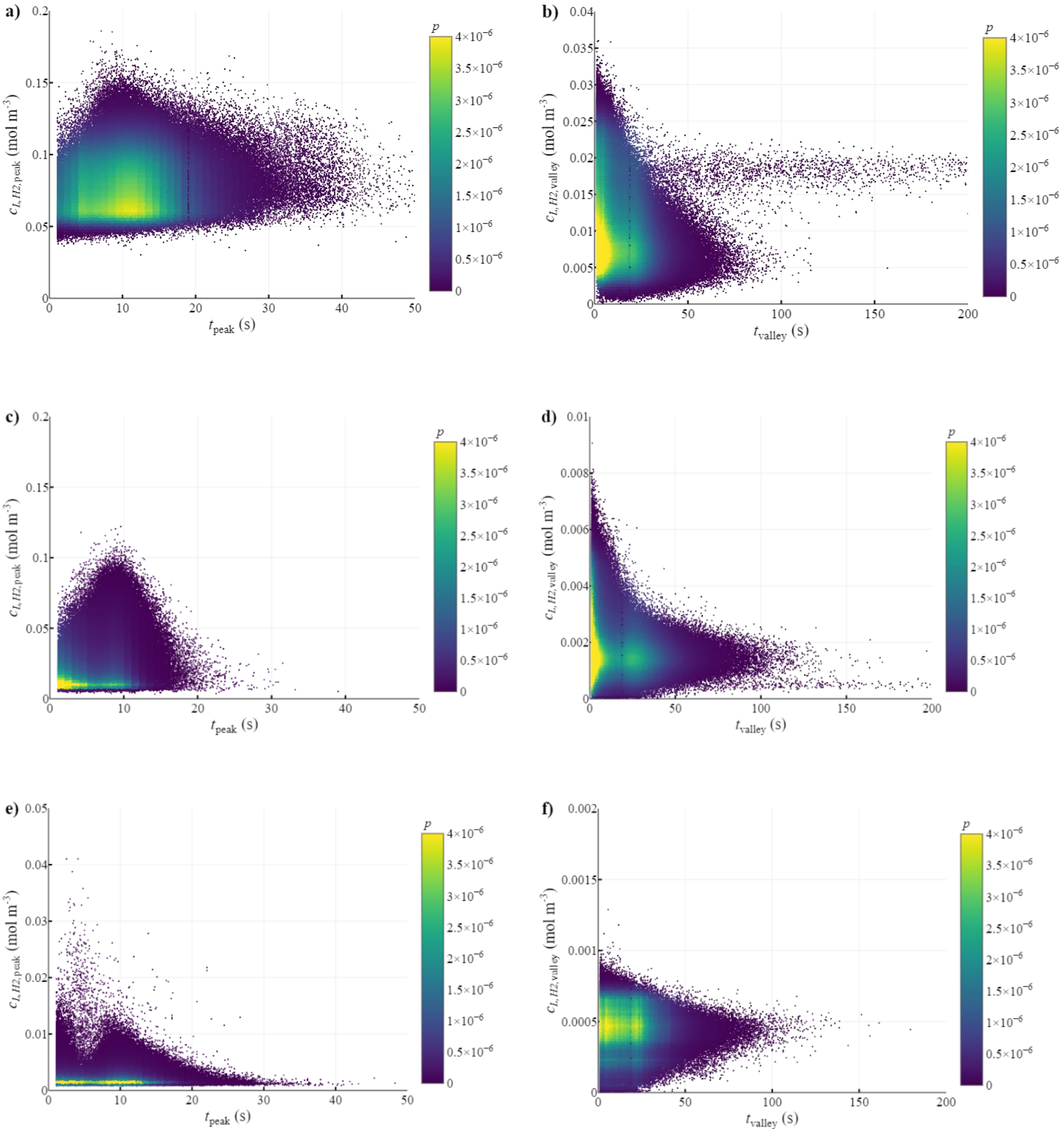

Figure S9. The probability of a microbe to experience a specific H_2 concentration peak or valley. Each dot represents a peak or valley with such a concentration and time, and is coloured by the probability of occurrence. Each row represents data obtained with a specific concentration: (a, b) 5, (c, d) 10 and (e, f) 25 g L^{-1}), with the peaks in the left column (a, c, e) and the valleys at the right (b, d, f).

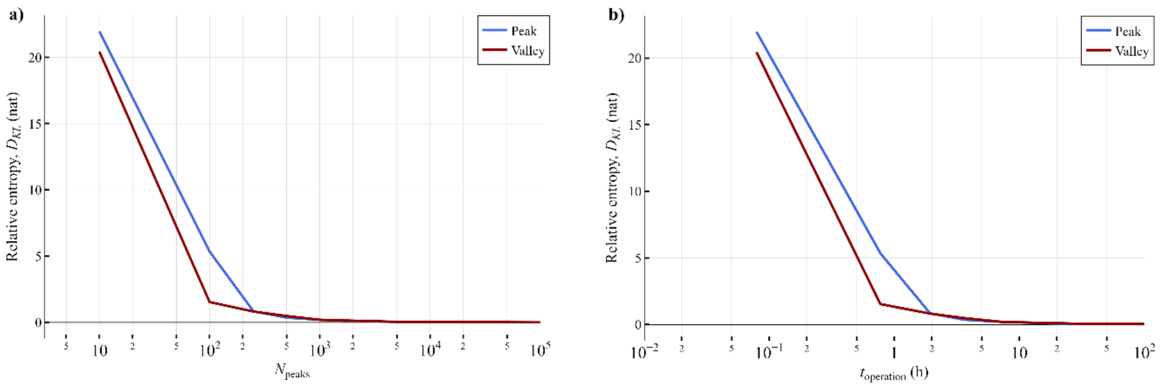

Figure S10. Minimization of the Kullback-Leibler divergence for a) the number of peaks used as input for the scale-down simulator, and b) the operational time of the scale-down simulator. D_{KL} was calculated by comparing the probability distribution of the residence time in the peak (blue line) or valley (red line) from the scale-down simulator with their respective CFD-derived counterpart. The probability distributions from the scale-down simulator were derived without assuming mass transfer and reaction.

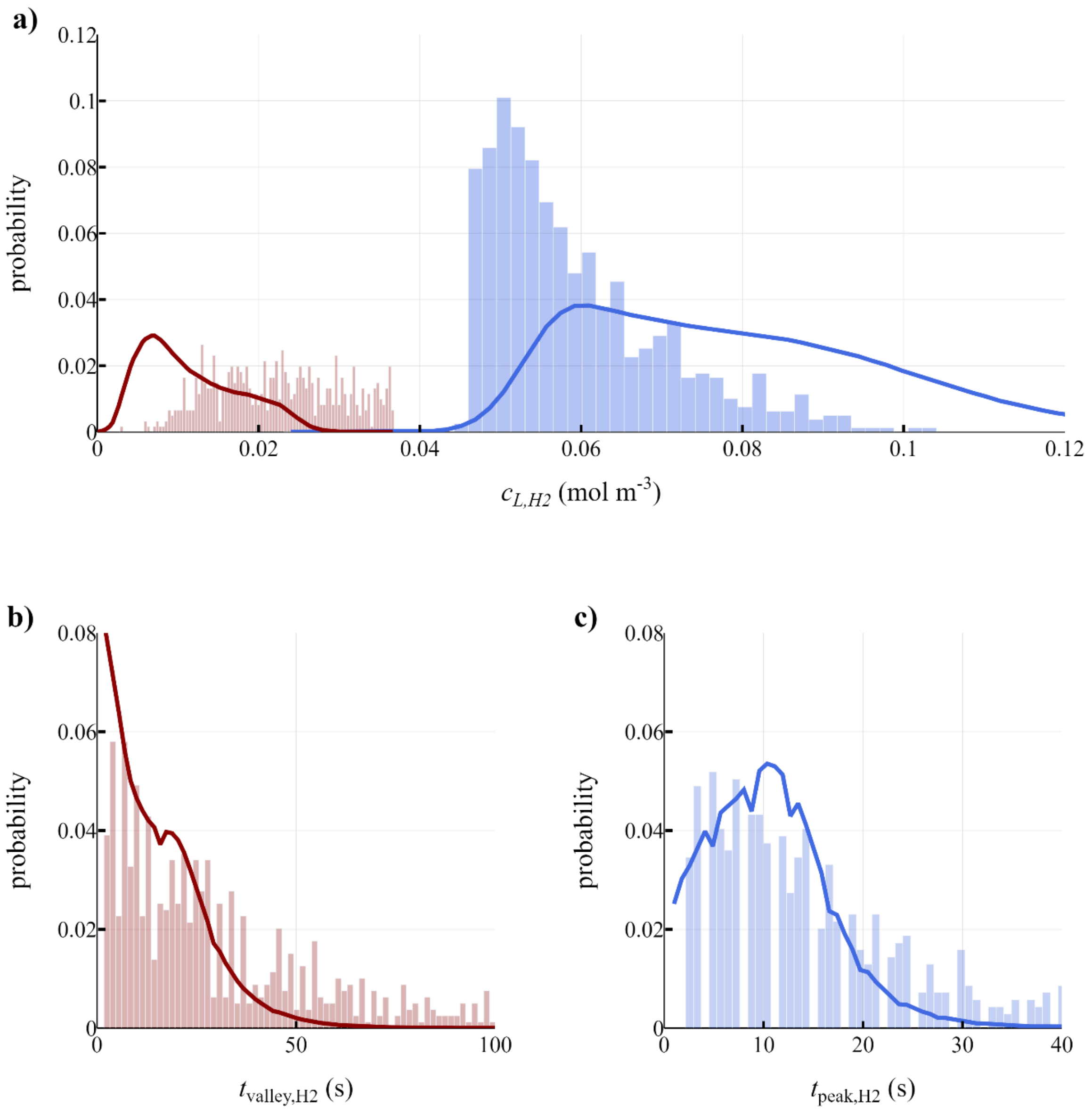

Figure S11. Comparison of the probability density functions obtained by the scale-down simulator (bars) with the CFD results (lines). Probability density functions for a) the concentration of dissolved H_2 during the peaks (blue) and the valleys (red), as well as the residence time in a b) valley or c) peak, obtained with 5 g L^{-1} biomass and simulating 2000 peaks.

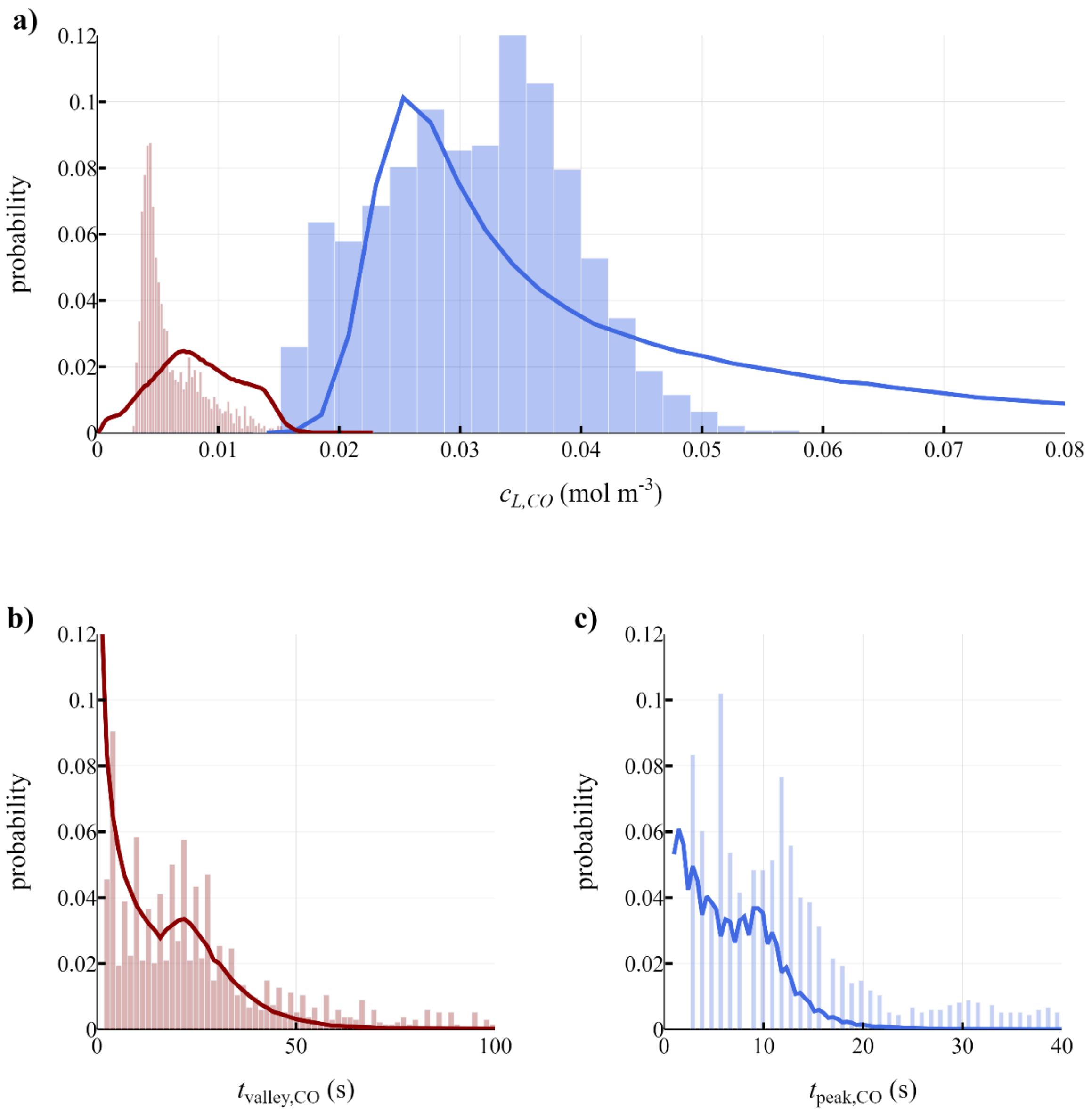

Figure S12. Comparison of the probability density functions obtained by the scale-down simulator (bars) with the CFD results (lines). Probability density functions for a) the concentration of dissolved CO during the peaks (blue) and the valleys (red), as well as the residence time in a b) valley or c) peak, obtained with 10 g L^{-1} biomass and simulating 2000 peaks.

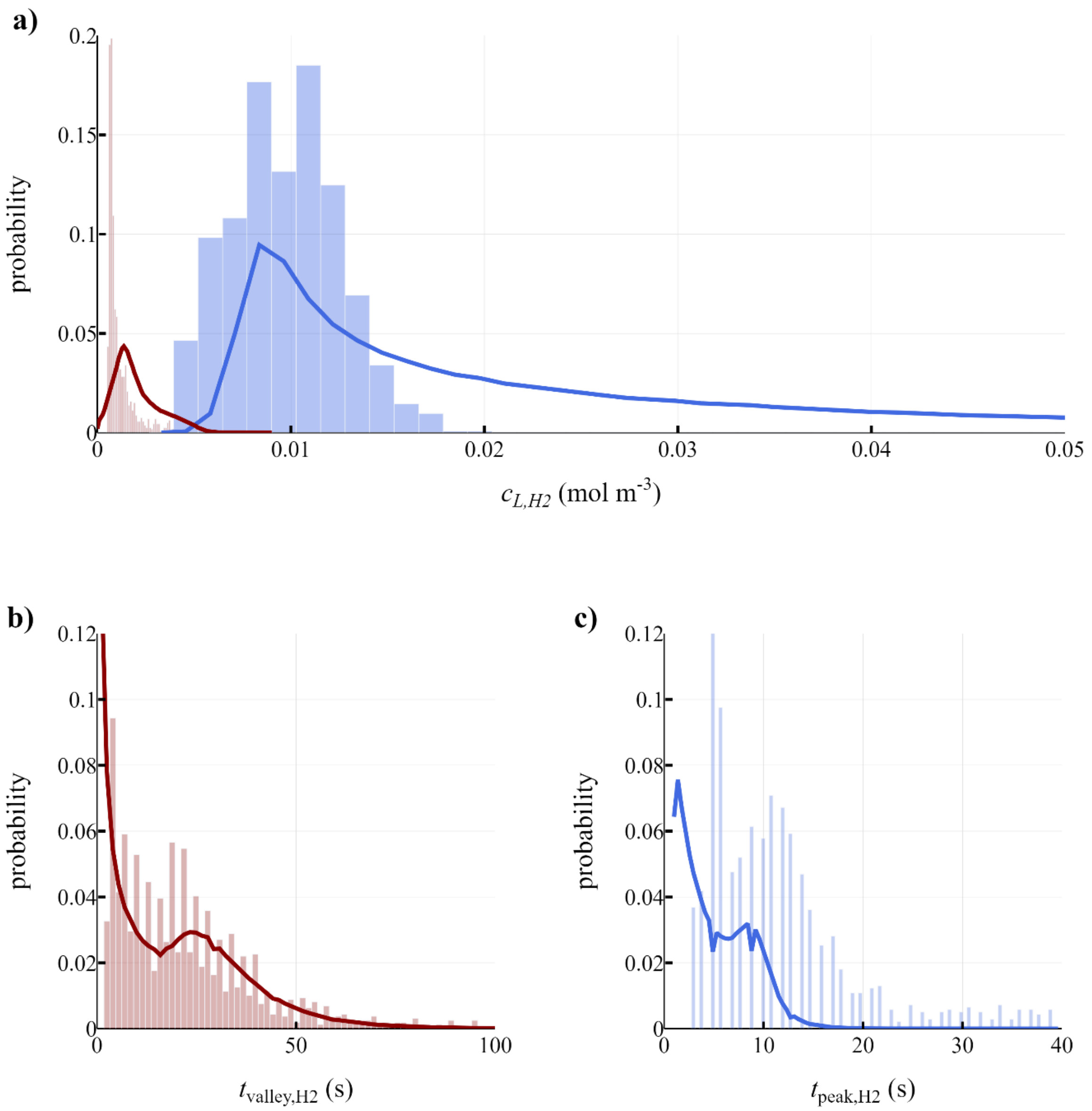

Figure S13. Comparison of the probability density functions obtained by the scale-down simulator (bars) with the CFD results (lines). Probability density functions for a) the concentration of dissolved H_2 during the peaks (blue) and the valleys (red), as well as the residence time in a b) valley or c) peak, obtained with 10 g L^{-1} biomass and simulating 2000 peaks.

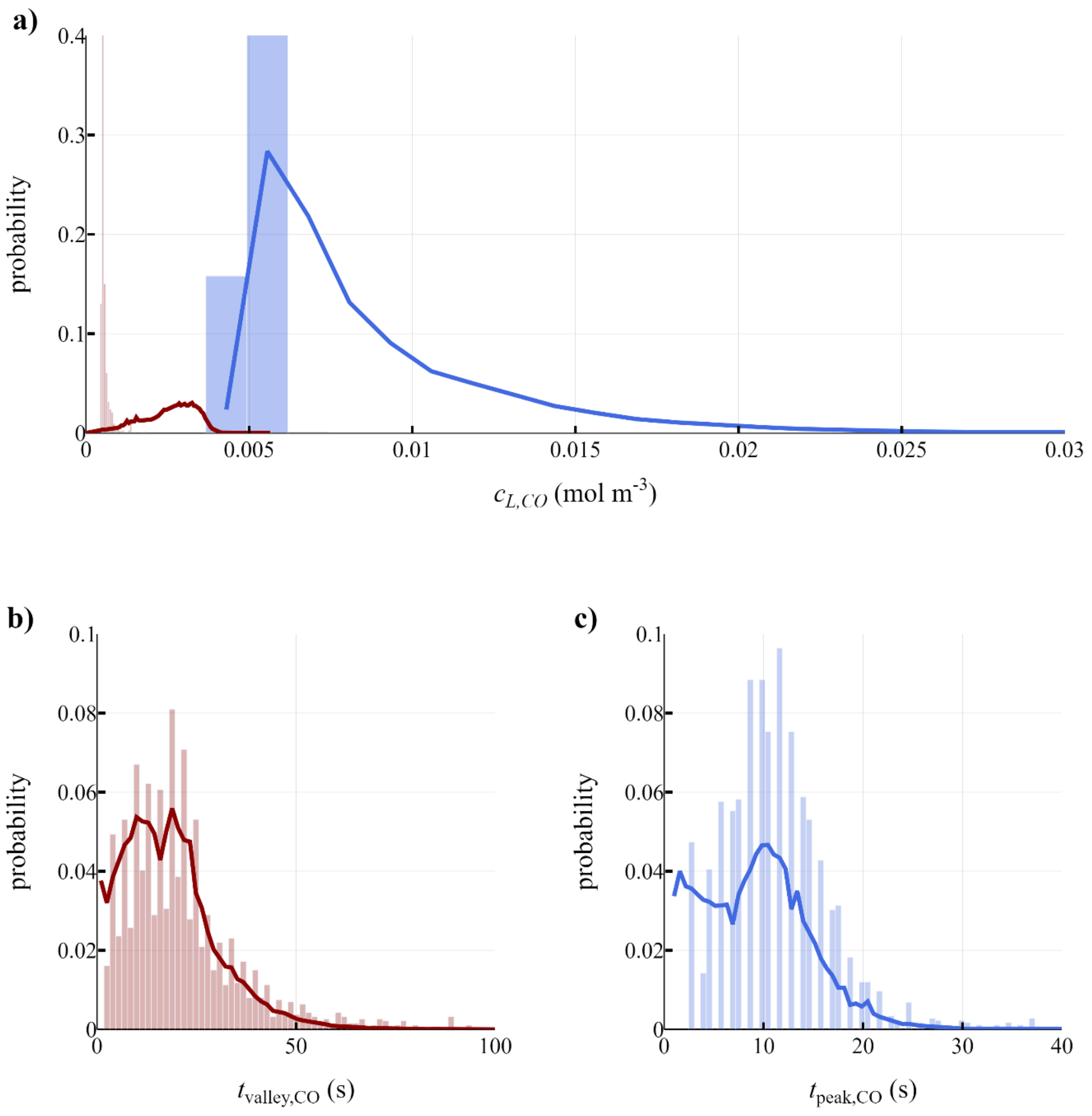

Figure S14. Comparison of the probability density functions obtained by the scale-down simulator (bars) with the CFD results (lines). Probability density functions for a) the concentration of dissolved CO during the peaks (blue) and the valleys (red), as well as the residence time in a b) valley or c) peak, obtained with 25 g L^{-1} biomass and simulating 2000 peaks.

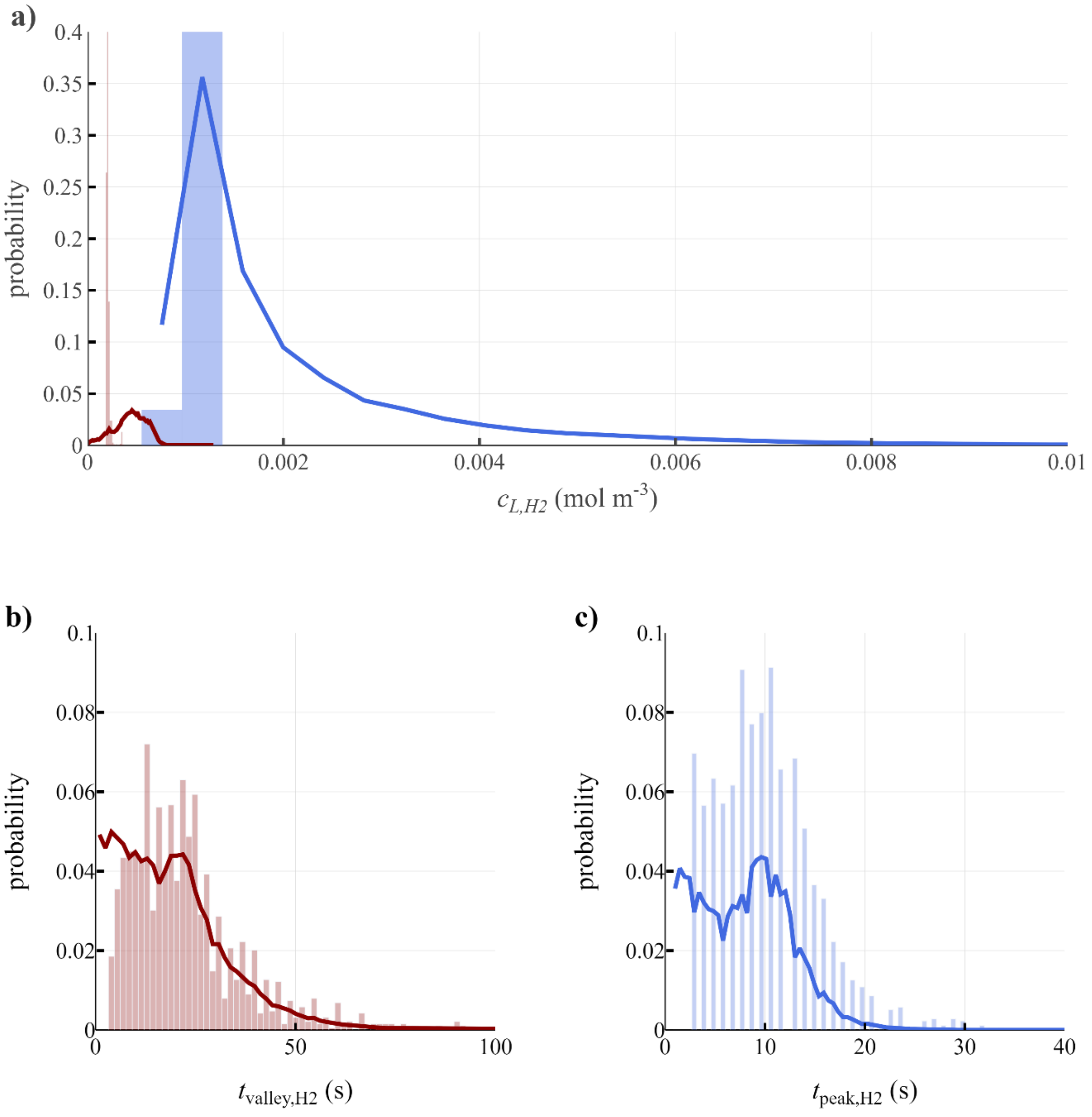

Figure S15. Comparison of the probability density functions obtained by the scale-down simulator (bars) with the CFD results (lines). Probability density functions for a) the concentration of dissolved H_2 during the peaks (blue) and the valleys (red), as well as the residence time in a b) valley or c) peak, obtained with 25 g L^{-1} biomass and simulating 2000 peaks.

Supplementary References

Akaike, H., 1998. Information theory and an extension of the maximum likelihood principle, in: Selected Papers of Hirotugu Akaike. Springer, pp. 199–213.

Bishop, C.M., 2006. Pattern Recognition and Machine Learning, 1st ed. Springer, New York.

Garcia-Ochoa, F., Gomez, E., 2009. Bioreactor scale-up and oxygen transfer rate in microbial processes: An overview. Biotechnol. Adv. 27, 153–176. https://doi.org/10.1016/j.biotechadv.2008.10.006

Haringa, C., Noorman, H.J., Mudde, R.F., 2017. Lagrangian modeling of hydrodynamic–kinetic interactions in (bio)chemical reactors: Practical implementation and setup guidelines. Chem. Eng. Sci. 157, 159–168. https://doi.org/10.1016/J.CES.2016.07.031

Haringa, C., Tang, W., Deshmukh, A.T., Xia, J., Reuss, M., Heijnen, J.J., Mudde, R.F., Noorman, H.J., 2016. Euler-Lagrange computational fluid dynamics for (bio)reactor scale down: An analysis of organism lifelines. Eng. Life Sci. 16, 652–663. https://doi.org/10.1002/elsc.201600061

Haringa, C., Tang, W., Wang, G., Deshmukh, A.T., van Winden, W.A., Chu, J., van Gulik, W.M., Heijnen, J.J., Mudde, R.F., Noorman, H.J., 2018. Computational fluid dynamics simulation of an industrial P. chrysogenum fermentation with a coupled 9-pool metabolic model: Towards rational scale-down and design optimization. Chem. Eng. Sci. 175, 12–24. https://doi.org/10.1016/j.ces.2017.09.020

Kuschel, M., Takors, R., 2020. Simulated oxygen and glucose gradients as a prerequisite for predicting industrial scale performance a priori. Biotechnol. Bioeng. 117, 2760–2770. https://doi.org/10.1002/BIT.27457

Lapin, A., Müller, D., Reuss, M., 2004. Dynamic behavior of microbial populations in stirred bioreactors simulated with Euler-Lagrange methods: Traveling along the lifelines of single cells. Ind. Eng. Chem. Res. 43, 4647–4656. https://doi.org/10.1021/IE030786K/ASSET/IMAGES/LARGE/IE030786KF00007.JPEG

McClure, D.D., Kavanagh, J.M., Fletcher, D.F., Barton, G.W., 2016. Characterizing bubble column bioreactor performance using computational fluid dynamics. Chem. Eng. Sci. 144, 58–74. https://doi.org/10.1016/J.CES.2016.01.016

Puiman, L., Abrahamson, B., van der Lans, R.G.J.M., Haringa, C., Noorman, H.J., Picioreanu, C., 2022. Alleviating mass transfer limitations in industrial external-loop syngas-to-ethanol fermentation. Chem. Eng. Sci. 259, 117770. https://doi.org/10.1016/J.CES.2022.117770

Siebler, F., Lapin, A., Hermann, M., Takors, R., 2019. The impact of CO gradients on C. ljungdahlii in a 125 m3 bubble column: Mass transfer, circulation time and lifeline analysis. Chem. Eng. Sci. 207, 410–423. https://doi.org/10.1016/j.ces.2019.06.018

van't Riet, K., van der Lans, R.G.J.M., 2011. Mixing in Bioreactor Vessels. Compr. Biotechnol. Second Ed. 2, 63–80. https://doi.org/10.1016/B978-0-08-088504-9.00083-0

Zanghi, A., Lin, D., Balsara, T., Young, L., Wolf, B., Huang, P., 2017. Increased efficiency and product quality with the UniVessel ® Single Use bioreactor for CHO fed-batch cultures.
