# Supplementary figures and images for: Downscaling Industrial-Scale Syngas Fermentation to Simulate Frequent and Irregular Dissolved Gas Concentration Shocks
Source: Bioengineering (Basel). 2023 Apr 25;10(5):518. doi: 10.3390/bioengineering10050518 (PMC10215885; doi:10.3390/bioengineering10050518)

## Slide 1
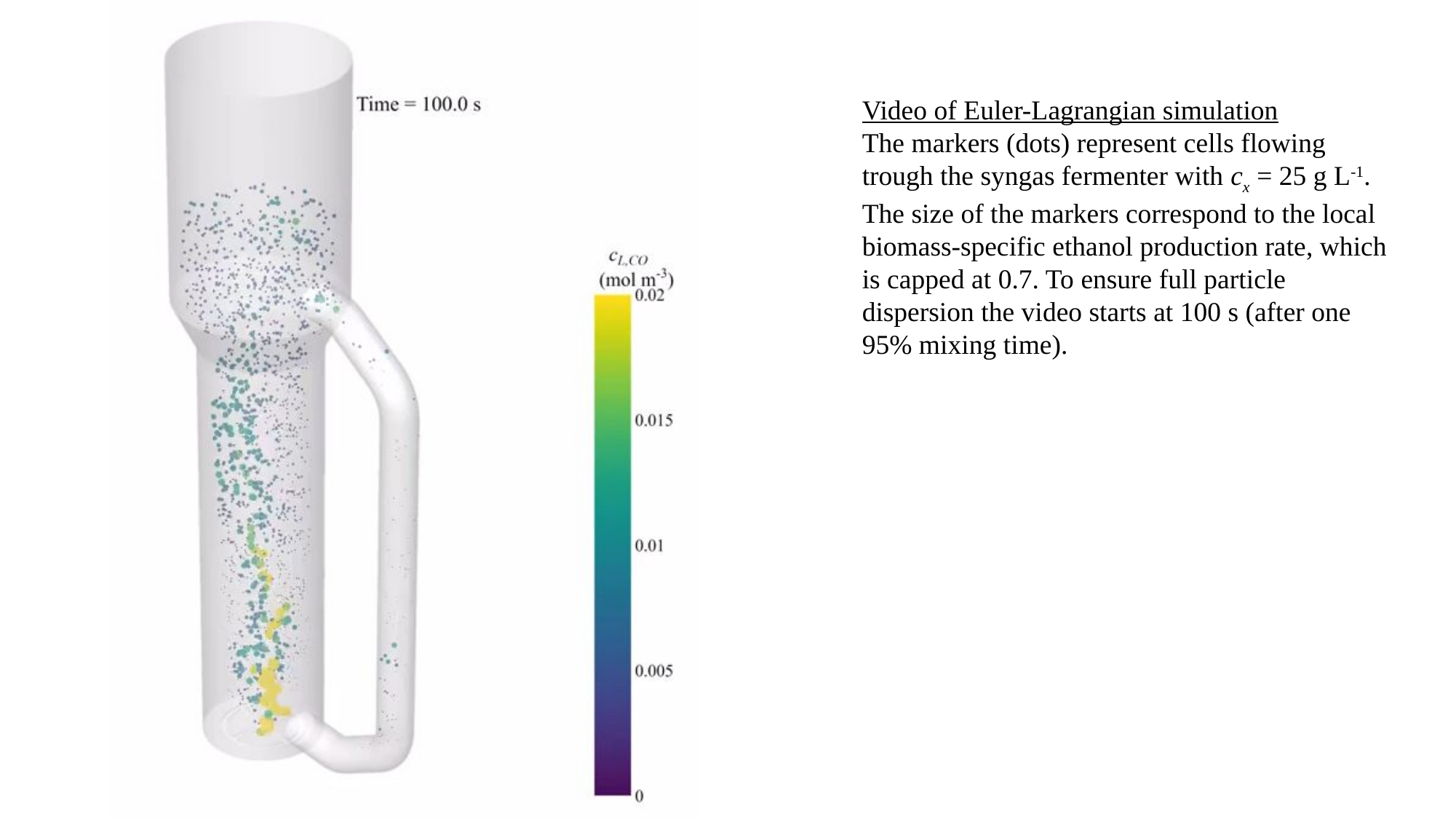

Supplement: Supplementary file 1 [file bioengineering-10-00518-s001.zip › VideoS1.pptx]
